# Pesticides and Ostreid Herpesvirus 1 Infection in the Pacific Oyster, *Crassostrea gigas*

**DOI:** 10.1371/journal.pone.0130628

**Published:** 2015-06-24

**Authors:** Pierrick Moreau, Nicole Faury, Thierry Burgeot, Tristan Renault

**Affiliations:** 1 Ifremer (Institut Français de Recherche pour l’Exploitation de la Mer), Unité Santé, Génétique et Microbiologie des Mollusques, Laboratoire de Génétique et Pathologie des Mollusques Marins, Ronce les Bains, 17390, La Tremblade, France; 2 Ifremer (Institut Français de Recherche pour l’Exploitation de la Mer), Ifremer Research Unit of Biogeochemistry and Ecotoxicology, rue de l’Ile d’Yeu, BP, 21105, 44311 Nantes, France; 3 Unité des Hépacivirus et Immunité Innée, Institut Pasteur, Paris, France; Bigelow Laboratory for Ocean Sciences, UNITED STATES

## Abstract

Since 2008, mass mortality outbreaks have been reported in all French regions producing Pacific oysters, and in several Member States of the European Union. These mass mortality events of Pacific oysters are related to OsHV-1 infection. They occur during spring and summer periods leaving suspect the quality of the marine environment and the role of seasonal use of pesticides associated with the arrival of freshwater in oyster rearing areas. Pesticides have been also detected in French coastal waters, especially in areas of oyster production. Using PMA real-time PCR we showed that a mixture of 14 pesticides has no effect on the integrity of virus capsids from viral suspension in the conditions tested. A contact of oysters with this pesticide mixture was related to higher mortality rates among experimentally infected animals in comparison with control ones (no previous pesticide exposure before experimental infection). We therefore suggest that pesticides at realistic concentration can exert adverse effects on Pacific oysters and causes an increased susceptibility to the viral infection in experimental conditions.

## Introduction

Since the early 90s, recurring mortality events have been associated with the detection of a herpesvirus in the Pacific oyster, *Crassostrea gigas* [1,2]. This virus named ostreid herpesvirus 1 (OsHV-1) can be considered one of the major infectious agents affecting farmed oysters in France. Since 2008, mass mortality outbreaks (spat and juvenile) have been reported in all French regions producing oysters, and in several Member States of the European Union. Available data suggest that OsHV-1 is a major cause with the detection since 2008 of a particular viral genotype, called μVar [3,4].

Pesticides have become more frequently detected among the pollutants found in estuarine and coastal areas [5–7]. Pesticides may have major ecological consequences and could endanger organism growth, reproduction or survival [8]. Many studies have been published concerning pollution and increased disease susceptibility for a variety of vertebrates [9–11] and some data are now also available for some invertebrates [12]. Pesticides are detected in French coastal waters, especially in oyster production areas. Pacific oysters filter large volumes of seawater and may accumulate or metabolize contaminants within their tissues [13,14]. The presence of pesticides in seawater appears thus an environmental risk for the Pacific oyster, *C*. *gigas*, already documented through previous works [12,15–17]. Mass oyster mortality outbreaks related to OsHV-1 infection occur during spring and summer periods [3,18] and can leave suspect the quality of the marine environment [5] and the role of seasonal use of pesticides associated with the arrival of freshwater in oyster farming areas[19]. The presence of pesticides in these areas could make oysters more vulnerable to infectious agents present in the environment, particularly OsHV-1.

In this context, the effects of a mixture of 14 pesticides at realistic concentrations were studied to determine whether the mixture could affect the development of OsHV-1 infection in Pacific oysters. In a first step, tests consisted of studying the effect of the mixed pesticides at different concentrations on viral suspensions during a 24 hours period. The effect of pesticides on the integrity of viral capsids was investigated using a propidium monoazide (PMA) real-time PCR. A viral suspension was also treated with the pesticide mixture prior injection in the adductor muscle of oysters in order to explore the effect of the pollutants on the capacity of the virus to induce infection and mortality. In a second step, the susceptiblity of Pacific oysters, *C*. *gigas*, to viral infection was investigated in experimental conditions, after a previous contact during 24 hours with the mixture of pesticides at two concentrations (1X and 10X). Oyster survival was monitored for the different conditions tested during 9 days post-infection. Oysters belonging to a family presenting moderate susceptibility to the viral infection were used in the present study in order to better appreciate effects of pesticide on OsHV-1 infection susceptibility.

## Materials and Methods

### 2.1 Pacific Oysters

Within the framework of the EU funded project Bivalife (FP7, 2011–2014), 45 bi-parental families of Pacific oysters, *Crassostrea gigas*, were produced in the Ifremer’s facilities located in La Tremblade (Charente Martime, France). These oyster families demonstrated contrasted susceptibility to different pathogens including OsHV-1. One of these families (F22) was selected on the basis of its moderate level (around 50% mortality rates), of susceptibility to OsHV-1 infection in experimental conditions. It was selected in order to better appreciate a possible adverse effect of pesticides on viral disease development and survival in experimentally infected animals. For this family, all animals tested were 11 months.

### 2.2 Pesticides

Fourteen pesticides belonging to 4 pesticide groups (herbicides, fongicides, insecticides and molluscicides) were selected based on their detection in the aquatic environment in Britany and Charente Maritime [20] and their immunotoxic potential for invertebrates. The mixture of the 14 selected pesticides consisted of carbaryl, fosetyl aluminium, alachlor, métolachlor, glyphosate, atrazine, terbuthylazine, diuron, AMPA, bentazon, tebuconazol, imidacloprid, mancozeb and metaldehyde [21] (Table 1). Solvents were used as recommended by manufacturers (Table 1) and final solvent concentration was less than 0.5% in order to avoid effects of the solvent. Pesticides were purchased from Fluka and Supelco (Sigma-Aldrich). It was decided to study the putative effects of pesticides using a mixture of 14 different molecules in order to better represent aquatic environments where a variety of substances may be simultaneously present. The effect of pesticide alone may be different when it is with other pesticides or chemical product mixture. Indeed, additive effects (sum of the effects of the molecules themselves), antagonists (reducing the effects of one or more products) or synergistic (one or more products increase greatly the effect of others) may occur for pesticide mixture.

**Table 1:** Final concentrations of the pesticide mixture and the solvents used.

Pesticides (solvent)	Groupe	Concentration μg/L (1x)*	Concentration μg/L (10x)	Concentration μg/L (200x)
Carbaryl (CH)	Insecticide	0.05	0.5	10
Fosetyl Al	Fongicide	0.6	6	120
Alachlor (MeOH)	Herbicide	0.8	8	160
Métolachlor (AN)	Herbicide	1	10	200
Glyphosate	Herbicide	4	40	800
Atrazine (MeTE)	Herbicide	0.1	1	20
Terbuthylazine	Herbicide	0.6	6	120
Diuron	Herbicide	2	20	400
AMPA	Herbicide	2.5	25	500
Bentazon	Herbicide	0.5	5	100
Tebuconazol	Fongicide	3	30	600
Imidacloprid (AN)	Insecticide	0.1	1	20
Mancozeb	Fongicide	0.1	1	20
Metaldehyde	Molluscicide	0.1	1	20

Solvents: CH. cyclohexane; MeOH. methanol; AN. acetonitrile; MeTE. methyl terbutyl ether

* environmentaly relevant concentrations

Unfiltered water samples (1000 mL of seawater from tanks) were analyzed to avoid sub-estimations in the total concentration by Multiresidue Pesticide Analysis by LC-MS/MS. We realized these analyzes to be sure to work with the right concentration of pesticides (true concentration corresponding to the theoretical concentration). Girpa corporate (Angers, France) performed the pesticides quantification.

### 2.3 Experimental Design

#### 2.3.1. In vitro

Assays were carried out to study the effect of the pesticide mixture *in vitro* on viral (OsHV-1) suspension [22]. Three concentrations of pesticide mixture were tested (1X, 10X and 200X) [21] with a 24 hour exposition at 15°C in the dark.

Quantification of non-broken capsid in viral suspension by PMA real-time PCR: PMA (phenanthridium,3-amino-8-azido-5-[3-(diethylmethylammonio)propyl]-6-phyenol dichloride; Biotium, Inc., Hayward, CA) was prepared in 20% dimethyl sulfoxide (DMSO) (Sigma-Aldrich Co., St. Louis, MO) to obtain a concentration of 20mM. The PMA stock solution was transferred to tubes that contained 280 μL of viral suspension at a final concentration of 500 μM. The same quantity of seawater (without PMA) was added to the viral suspension for the negative control. Light-transparent 1.5-ml microcentrifuge tubes were used. All manipulations of PMA solution were performed under minimal light to prevent any potential chemical change in PMA structure, as it is a light-sensitive molecule. Following 10 min of incubation in the dark with agitation (600 rpm), samples were exposed for 10 min to Phast Blue light [23].

DNA extraction from viral DNA suspension with and without PMA treatment was carried out using DNA Extraction Kit QIAmp (Qiagen) under minimal light. OsHV-1 DNA quantification was performed as described below (cf. OsHV-1 DNA quantification by real-time PCR). The percentages of viral non-broken capsids were defined as the a ratio: OsHV-1 DNA quantification with PMA / OsHV-1 DNA quantification without PMA.

#### 2.3.2. In vivo

**Fig 1:**
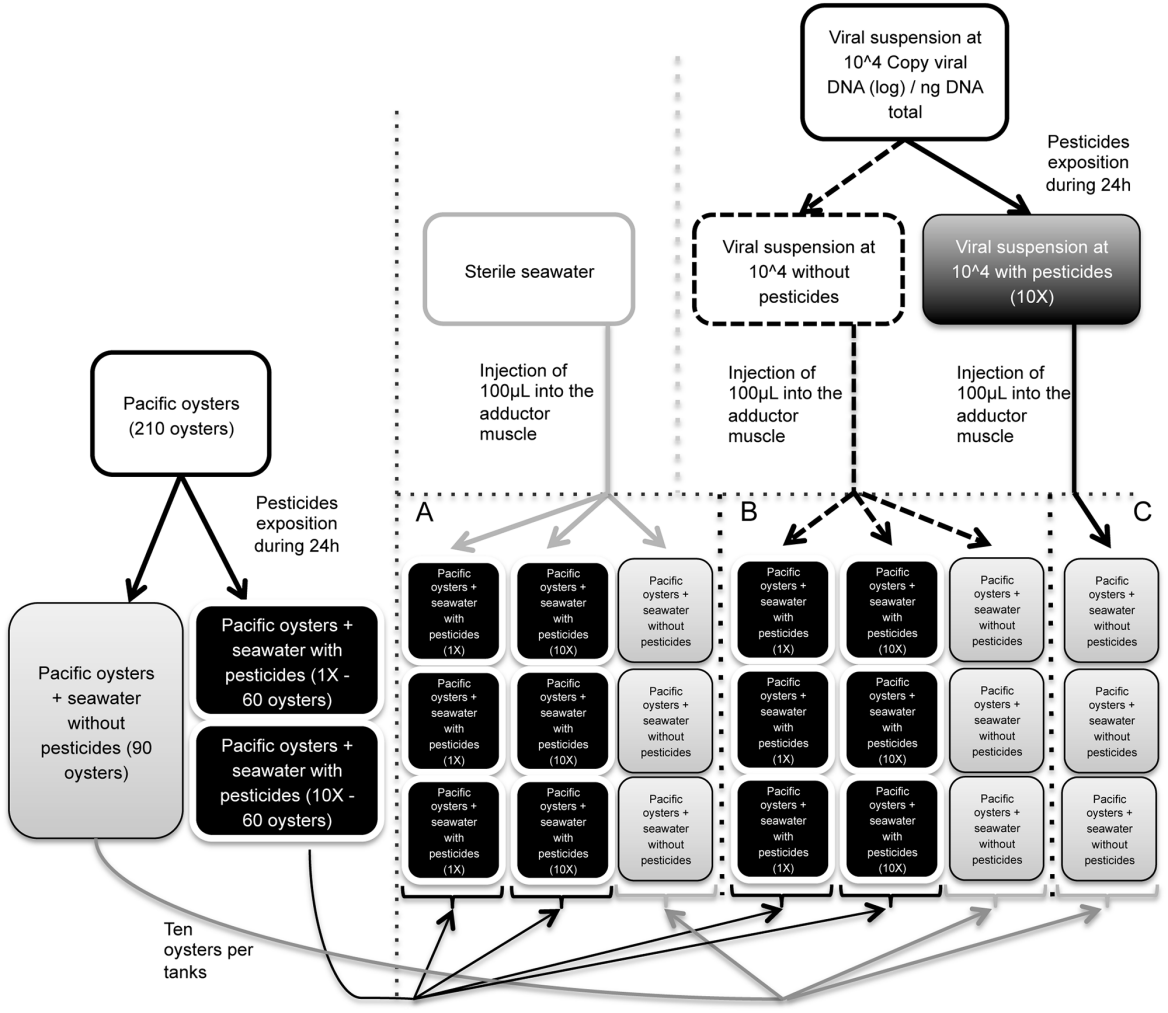
Design of pesticide exposition and experimental viral infection. Pesticide contamination. Pacific oysters were exposed with the mixture of 14 pesticides during 24h. Oysters were not fed during the pesticide contamination. Three tanks were used in each experiment: 2 tanks with pesticides (60 oysters per tank, 120 oysters in total) and 1 tank without pesticides (90 oysters) receiving the same quantity of solvent used for preparing pesticides (the solvent was added in the water; negative controls). Experimental viral infection. After pesticide exposition during 24 hours, experimental OsHV-1 infection was carried out by intramuscular injection of a viral suspension. Experimental design included also oysters injected with an OsHV-1 suspension treated with pesticides. Two hundred and ten oysters were “anaesthetized” during 4 h. One hundred μL of an OsHV-1 (μVar genotype) suspension at 1 × 10^4^ copies of viral DNA/μL or sterile artificial water were injected. Oysters were then placed in tanks containing 3 L of filtered seawater (1 μm) at 22°C without food supply (ten oysters per tank) using the following design: (A) oysters injected with sterile artificial seawater (3 tanks with seawater for oysters without pesticides, 6 tanks with seawater for oysters with pesticide exposure), (B) oysters injected with OsHV-1 suspension (3 tanks with seawater for oysters without pesticides, 6 tanks with seawater for oysters with pesticide exposure), (C) oysters injected with OsHV-1 suspension treated with pesticides 10X (3 tanks with seawater for oysters with pesticide exposure). Survival was monitored during nine days after injection.

Pesticide contamination: Pacific oysters were exposed to the mixture of 14 pesticides (1X and 10X) for 24h. Oysters were not fed during the pesticide contamination. Temperature of seawater in tanks was maintained between 19.5°c and 20°C. Three tanks were used in each experiment: 2 tanks with pesticides (60 oysters per tank, 120 oysters in total) and 1 tank without pesticides (90 oysters) receiving the same quantity of solvent used for preparing pesticides (the solvent was added in the water; negative controls) (Fig 1).

Experimental viral infection: After pesticide exposure for 24 hours, experimental OsHV-1 infection was carried out by intramuscular injection of a viral suspension. The experimental design also included oysters injected with an OsHV-1 suspension treated with pesticides. Two hundred and ten oysters were “anaesthetized” during 4 h [22]. One hundred μL of an OsHV-1 (μVar genotype, [3,4]) suspension at 1 × 10^4^ copies of viral DNA/μL or sterile artificial water were injected. Oysters were then placed in tanks containing 3 L of filtered seawater (1 μm) at 22°C without food supply (ten oysters per tank) using the following design (Fig 1):

1. oysters injected with sterile artificial seawater (3 tanks with seawater for oysters without pesticides, 6 tanks with seawater for oysters with pesticide exposure)
2. oysters injected with OsHV-1 suspension (3 tanks with seawater for oysters without pesticides, 6 tanks with seawater for oysters with pesticide exposure)
3. oysters injected with OsHV-1 suspension treated with pesticides 10X (3 tanks with seawater for oysters with pesticide exposure).

Survival was monitored during nine days after injection. Percentages of cumulative survival were defined daily for the different conditions. Dead oysters were removed from tanks during the time course of the experiment. The experiment was performed three times.

### 2.4 DNA Extraction

Total DNA was extracted from mantle fragments using QiAamp tissue mini kit (QIAgen) combined with the use of the QIAcube automate, according to the manufacturer's protocol. Elution was performed in 200 μL of AE buffer provided in the kit. The DNA quality and quantity were determined using NanoDrop 2000 (Thermo Scientific). Extracted DNA was stored at -20°C for OsHV-1 DNA quantification by real-time PCR or 4°C for non-broken capsid quantification by PMA real-time PCR.

### 2.5 OsHV-1 DNA Quantification by Real-Time PCR

OsHV-1 DNA quantification was carried out using a real-time PCR protocol [24]. Real-time PCR was performed in duplicate using a Mx3000 Thermocycler sequence detector (Agilent). Amplification reactions were performed in a total volume of 20 μL. Each well contained 5 μL of genomic DNA (5 ng/μL), 10 μL of Brillant III Ultra-Fast SYBR Green Master Mix (Agilent), 2 μL of each primer (5 μM: OsHVDPFor 5’ATTGATGATGTGGATAATCTGTG3’; and 5 μM OsHVDPRev 5’GGTAAATACCATTGGTCTTGTTCC 3’;[25]) and 1 μL of distilled water. Real-time PCR cycling conditions were as follow: 3 min at 95°C, followed by 40 cycles of amplification at 95°C for 5 s, 60°C for 20 s. The results were expressed as a log10 of the virus DNA copy number per ng of total DNA.

### 2.6 Statistical Analysis

Statistical analysis was performed using the Wilcoxon—Mann Whitney test by statistical software R, to compare two groups. The Kruskal-Wallis test was used to compare more than two groups. The null hypothesis (H0) was that the distribution of the quantitative variable was the same in the groups. Significance was set at p ≤ 0.05 (*) and at p ≤ 0.01 (***). This test was used for all experiments.

LogRank test and Wilcoxon—Mann whitney tests were performed to compare survival oysters between the differents conditions. Significance was set at p ≤ 0.05 (*) and at p ≤ 0.01 (***).

## Results

### 3.1 In Vitro

**Fig 2:**
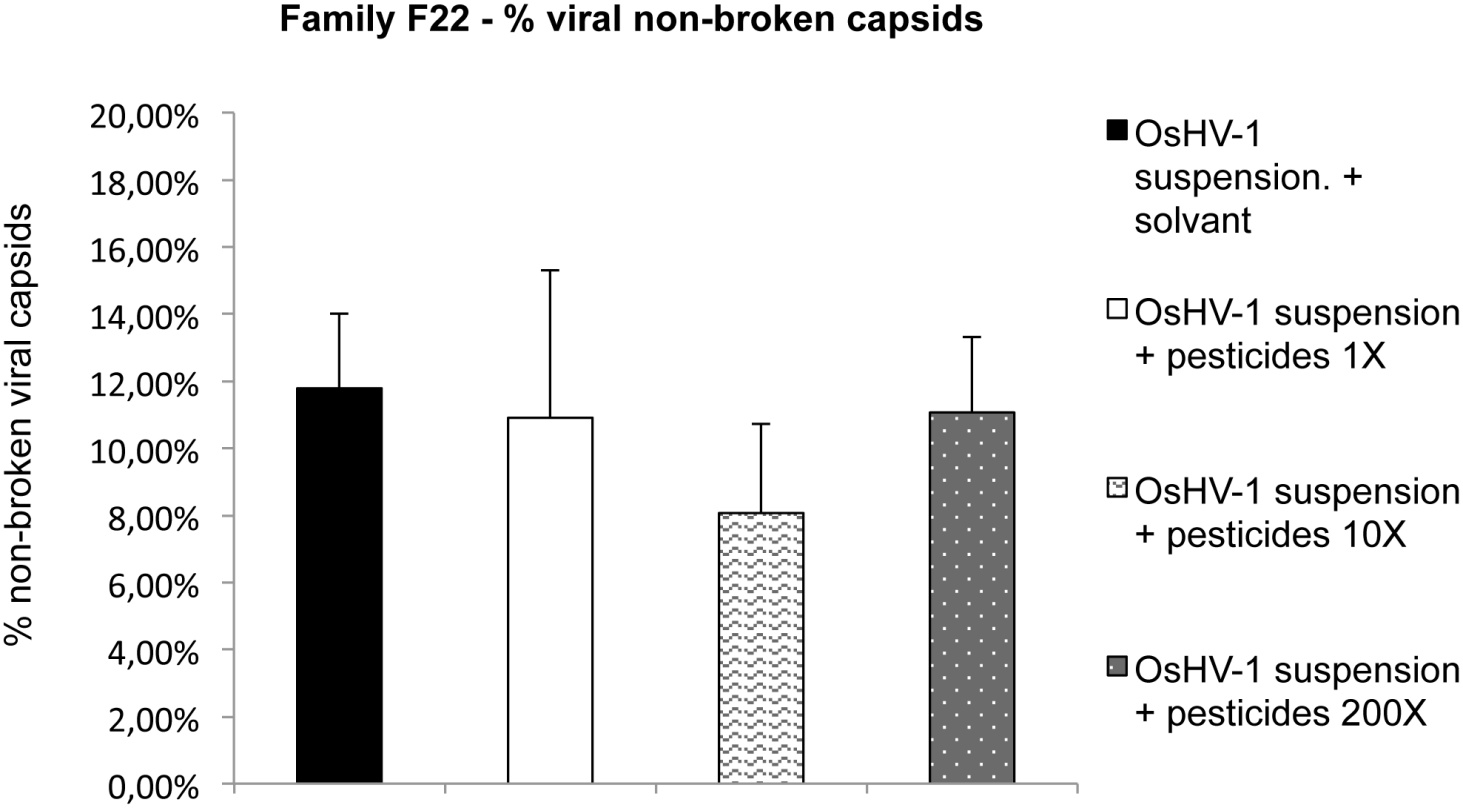
Percentages of viral non-broken capsids. Three concentrations of pesticide mixture were tested (1X, 10X and 200X) with a 24 hour exposition at 15°C in the dark. The percentages of viral non-broken capsids were defined as the a ratio: OsHV-1 DNA quantification with PMA / OsHV-1 DNA quantification without PMA. Error bars represent ± standard deviation. The experiment was performed three times (n = 12 samples for each conditions).

No significant effect of the pesticide mixture on the integrity of OsHV-1 capsids from the viral suspension was observed during the study regardless of the concentration of the pesticide mixture used (1X, 10X and 200X). Real-time PCR assays carried out in presence and absence of PMA did not demonstrate significant differences (Fig 2). The percentage of viral non-broken capsids was 12% for viral suspension without pesticides (Fig 2). For viral suspensions exposed to the pesticides mixture for 24 hours, the percentage of viral non-broken capsids was 11,4% for OsHV-1 suspension exposed to the pesticides mixture at 1X (Fig 2), 8,02% for OsHV-1 suspension exposed to the pesticides mixture at 10X (Fig 2), and 11,78% for OsHV-1 suspension exposed to the pesticides mixture at 200X (Fig 2).

### 3.2 In Vivo

**Fig 3:**
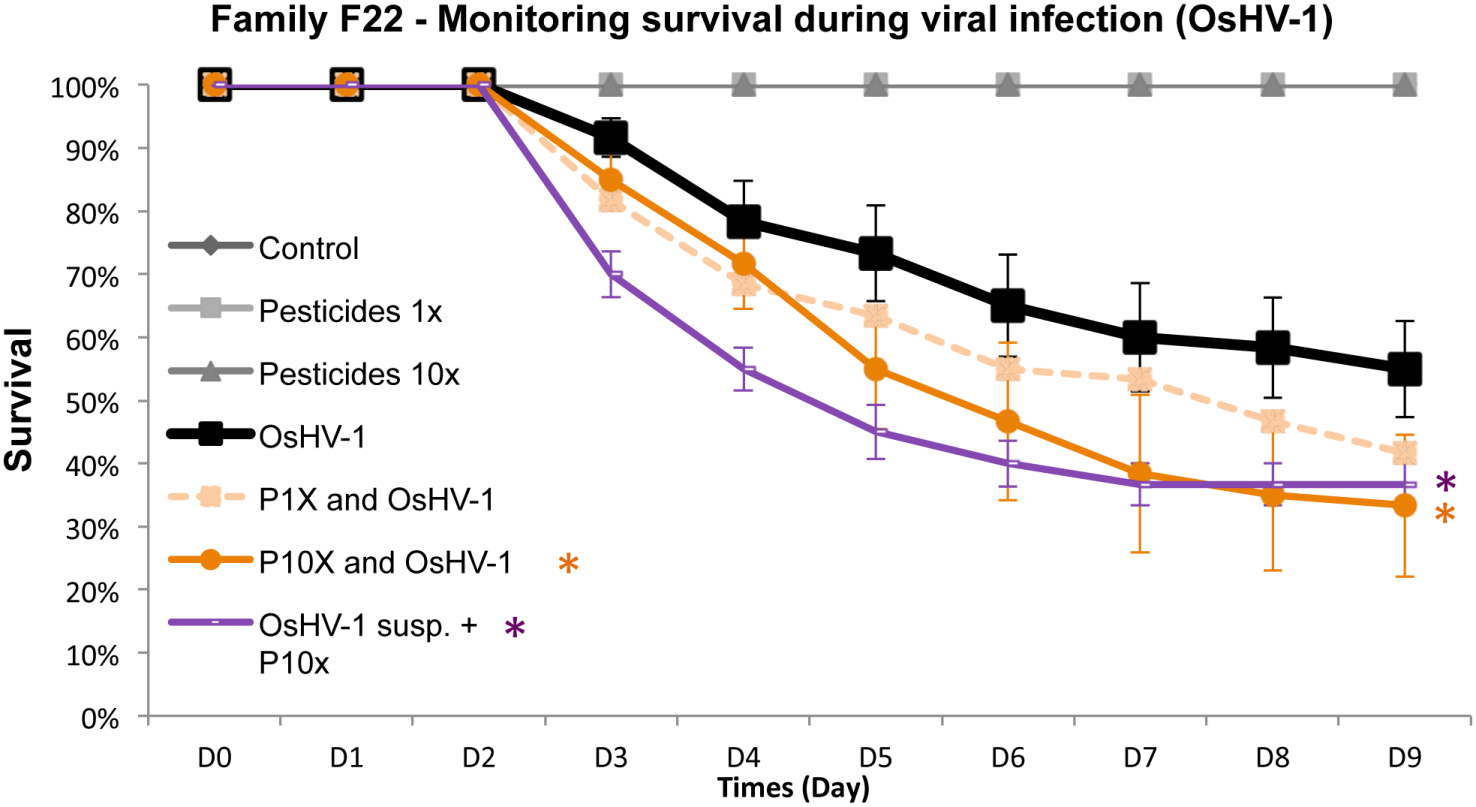
Survival (%) monitoring during experimental viral infection (OsHV-1) after exposure to the mixture of pesticides for 24h. In oyster family F22, Survival was monitored during nine days after injection. Percentages of cumulative survival were defined daily for the different conditions. Dead oysters were removed from tanks during the time course of the experiment. 100% of survival was reported among negative controls injected with 100 μL of artificial seawater (Control, Pesticides 1x and Pesticides 10x). Error bars represent ± standard deviation. The experiment was performed three times (n = 90 samples for each conditions).

Higher mortality rates were observed for Pacific oysters experimentally infected (injected with the viral suspension without previous contact with pesticides) after a 24 h exposure to the 14 pesticide mixture regardless of concentration (Fig 3). Mortality increased with pesticide exposure when animals were also exposed to the OsHV-1 compare to animals exposed to the OsHV-1 without pesticide exposure. From 5 days post-infection to 9 day post-infection, significantly higher mortality rates were observed for animals previously exposed to pesticides at the highest concentration (Fig 3: P10X and OsHV-1). After an oyster contact with the pesticides mixture at both concentrations tested (Table 2. 1X and 10X), no mortality was observed for oysters receiving an injection of sterile seawater (Fig 3: Pesticides 1X and Pesticides 10X).

**Table 2:** Theoretical and true concentrations of the pesticide mixture at 0h and 24h after pesticides exposure .

	Theoretical concentration		True concentration (0h)		True concentration (24h after addition of pesticides)	
Pesticides (solvent)	Concentration μg/L (1x)	Concentration μg/L (10x)	Concentration μg/L (1x)	Concentration μg/L (10x)	Concentration μg/L (1x)	Concentration μg/L (10x)
Carbaryl (CH)	0,05	0,5	< ld	0,95	< ld	0,465
Fosetyl Al	0,6	6	NQ	NQ	NQ	NQ
Alachlor (MeOH)	0,8	8	0,875	11,5	0,75	8
Métolachlor (AN)	1	10	2,65	21	1,925	14,25
Glyphosate	4	40	NQ	NQ	NQ	NQ
Atrazine (MeTE)	0,1	1	0,135	0,725	< ld	0,575
Terbuthylazine	0,6	6	0,53	2,555	0,3275	4,525
Diuron	2	20	1,5	14,75	1,325	15,75
AMPA	2,5	25	NQ	NQ	NQ	NQ
Bentazon	0,5	5	0,36	4,325	0,35	3,075
Tebuconazol	3	30	3,425	35,5	2,8	32,5
Imidacloprid (AN)	0,1	1	0,295	5	0,315	4,2
Mancozeb	0,1	1	NQ	NQ	NQ	NQ
Metaldehyde	0,1	1	NQ	NQ	NQ	NQ

Solvents: CH, cyclohexane; MeOH, methanol; AN, acetonitrile; MeTE, methyl terbutyl ether

ld: detection limit

NQ: not quantified

Higher mortality rates were also reported for oysters experimentally infected through an injection of a virus supension previously treated with the pesticides mixture at the highest concentration tested (10X) before injection into the adductor muscle (Fig 3: OsHV-1 susp. + P10X). Regarding the lower concentration (1X) of the pesticide mixture, no significant difference in terms of oyster survival was observed between the different conditions (Fig 3: P1X and OsHV-1), but mortality rates were intermediate to”OsHV-1” and”P10X and OsHV-1” (or”OsHV-1 susp. + P10X”) (Fig 3).

**Fig 4:**
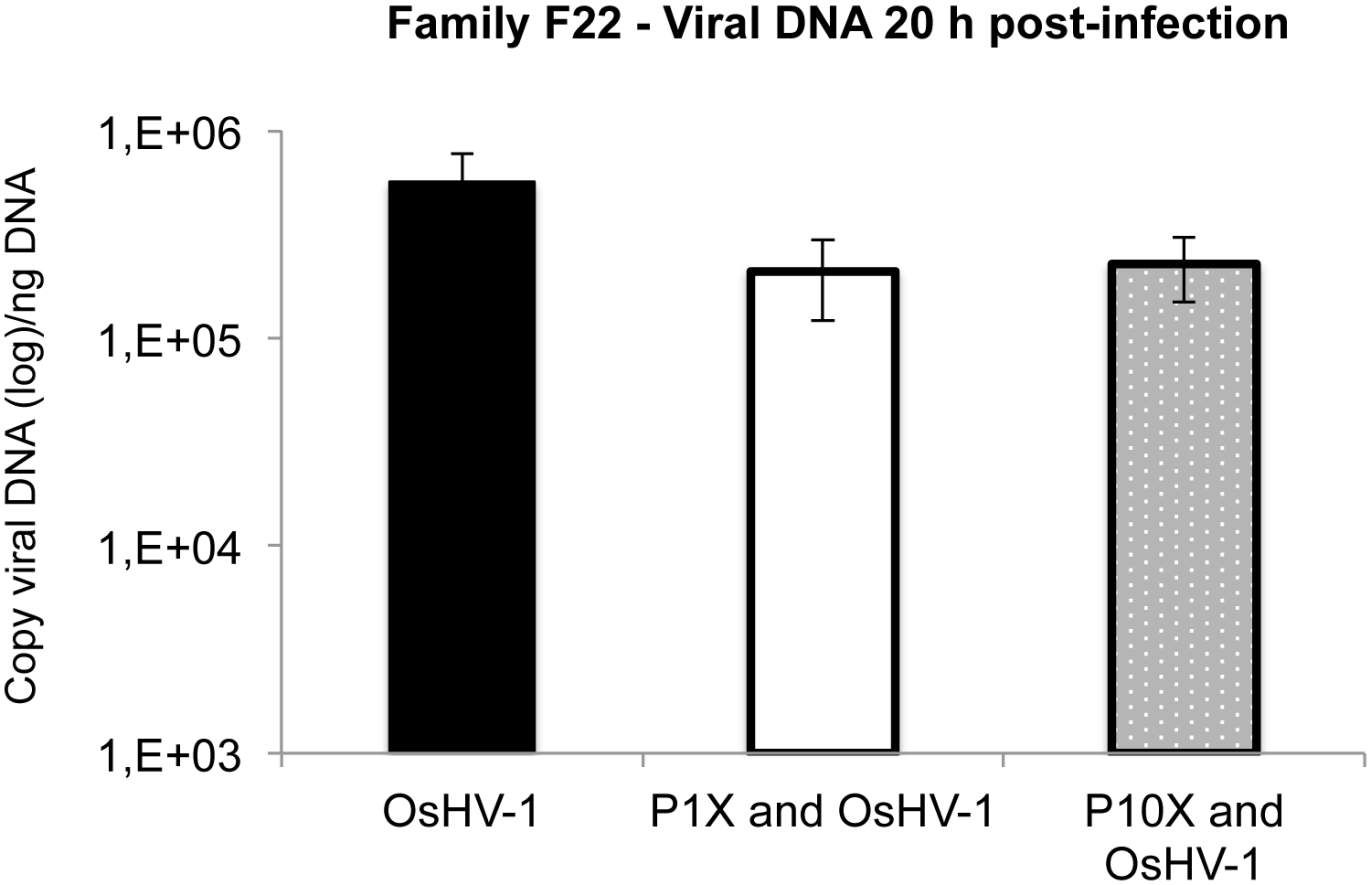
Viral DNA quantification 20h post-infection. In oyster family F22, a result of 0 DNA copies/ng of total DNA was reported for negative controls injected with 100 μL of artificial seawater. Error bars represent ± standard deviation. The experiment was performed three times (n = 90 samples for each conditions).

OsHV-1 DNA was quantified 20 h post infection. No significant differences were observed between conditions (Fig 4). For condition OsHV-1 without pesticides, the viral DNA was 5.6 10^5^ viral DNA copies per ng of total DNA at 20 h post-infection (Fig 4: OsHV-1). For condition OsHV-1 with pesticides exposure, the viral DNA was 2.1 10^5^ viral DNA copies per ng of total DNA at 20 h post-infection for the lower concentration of pesticides (Fig 4: P1X and OsHV-1). The viral DNA was 2.3 10^5^ viral DNA copies per ng of total DNA at 20 h post-infection for the higher concentration of pesticides (Fig 4: P10X and OsHV-1).

## Discussion

In a first step, direct effects of the pesticide mixture on the virus itself were explored. The tests consisted of studying potential effects of pesticides on viral suspensions in *in vitro* assays using 3 pesticide mixture concentrations (1X, 10X and 200X). Pesticide effects on the integrity of virus capsids were investigated by PMA real-time PCR. Propidium Monoazide (PMA) is a photo-reactive dye that preferentially binds to double-stranded DNA [23,26]. Only broken viral capsids allow PMA penetration and DNA binding. Blue light illumination (Fast blue apparatus) induces a photochemical reaction that leads to covalent binding of viral DNA and PMA. PMA binding to viral DNA makes its non-amplifiable by real-time PCR. In our study, OsHV-1 DNA contained in broken capsids could bind to PMA and was not amplifiable. OsHV-1 DNA from non-broken capsids failed to bind covalently DNA. Thus, after treatment with PMA, only viral DNA from non-broken capsids was amplifiable by real-time PCR. This technique is designed to discriminate viable pathogens and infectious ones (eg bacteria or viruses) [27–30]. No effect of the 14 pesticide mixture at the 3 concentrations tested was observed on the integrity of virus capids. This result suggested that the pesticides used in the present study were not able to brake OsHV-1 capsids after a 24 h contact in the conditions tested.

Although the methodological approach used (PMA real-time PCR) allowed us to appreciate potential effects on the viral capsid as broken capids are PMA permeable [27,28], it failed to identify effects on the whole viral particle including the viral envelop. The envelop is an essential structure for the entry of the virus in host cells [31–34] and modifications of the viral envelop can result in reduced infectivity. It has been previously reported that pesticides can induce modifications of cell membranes [35,36]. In order to explore such effects on the OsHV-1 envelop, a viral suspension was treated with the pesticide mixture prior to injection in the adductor muscle of oysters. This approach did not allow to observe a decrease of mortality rates suggesting that the pesticide mixture has no effect on the viral envelop and OsHV-1 infectivity. In tested conditions, the mixture of pesticides did not induce direct effects on viral particles.

The decrease in oyster survival upon OsHV-1 infection after a previous contact with the pesticide mixture could be thus associated with pesticide effects on Pacific oysters themselves. Under experimental conditions, a 24 hours contact with the mixture of 14 pesticides was related to increased mortality rates that could be interpreted as an increased susceptibility of Pacific oysters to OsHV-1 infection. A previous study showed that a mixture of 8 pesticides at realistic concentrations caused a reduction of haemocyte phagocytic activity in the Pacific oyster, as well as an increased susceptibility to bacterial infection in laboratory conditions [12]. We therefore suggest that the mixture of 14 pesticides induced adverse effects on the immune system of oysters, allowing the viral infection to increase more rapidly. Effects of pesticides on the expression of genes related to immunity were previously reported in Pacific oysters [12,37]. Relationships between pollution and an increased susceptibility towards infectious diseases have been established in several vertebrates [38–44]. Mercury chloride aggravates infection with HSV-2 virus in mice by increasing the replication and spread of the virus [45]. In the frog, *Rana pipiens*, exposure to a mixture of six pesticides decreased lymphocytes T cell proliferation and these immunocompromised animals exhibited an increase in the prevalence of a trematode parasite [46]. Another study also showed a decrease in amphibian survival linked to the presence of a pesticide mixture and a viral disease [42]. Finally, atrazine significantly increased susceptibility of salamender larvae to viral infection [47]. Few studies attempted to link contaminants and susceptibility to pathogens in marine molluscs [12,48–51]. A study reported that mortality rates of *Meretrix lusoria* were higher when animals was exposed to copper, cadmium, mercury or zinc during infection by birnavirus, compared to animals exposed to the virus alone [52].

Pacific oysters are sessile bivalve molluscs. They appear to be organims well suited to provide information upon the quality of coastal waters. Nowadays, estuaries are among the most contaminated areas and pesticides are detected in these areas especially during spring and summer periods. Thus, it is important to take into account possible interactions between oysters, pesticides and environmental factors in order to explain mass mortaltity outbreaks. It appears of great interest to compare transcriptomes based on RNAseq to better understand interactions between pesticidies, the Pacific oyster immune system and pathogens. Publication of the Pacific oyster genome [53] will also help greatly the study of genes involved in immunity. Although pesticides seems not able to direclty kill oysters, they must be considered carefully as they can be additional stress factors that render oysters more susceptible to pathogens and massive mortality outbreaks.
